# Infliximab-induced lupus with recurrent pericarditis: longitudinal CMR assessment of inflammatory resolution

**DOI:** 10.1007/s10554-026-03639-3

**Published:** 2026-03-16

**Authors:** Vaidehi Mendpara, Joseph El Roumi, Allan Klein

**Affiliations:** 1 Department of Internal Medicine, Cleveland Clinic, Cleveland, OH US; 2 Department of Cardiovascular Imaging, Heart, Vascular and Thoracic Institute, Cleveland Clinic, Cleveland, OH US

**Keywords:** Drug Induced Lupus Erythematosus [DILE], Infliximab, Recurrent pericarditis, Cardiac magnetic resonance, Interleukin-1 inhibitor [Anakinra], Pericardial inflammation

## Abstract

Recurrent pericarditis is an uncommon extraintestinal manifestation of inflammatory bowel disease and may arise from biologic-induced autoimmunity. Anti–tumor necrosis factor (TNF) agents such as infliximab can trigger drug-induced lupus, with pericardial involvement representing a rare presentation. Cardiac magnetic resonance (CMR) enables precise evaluation of pericardial inflammation, guiding treatment strategies and supporting safe immunomodulatory tapering.

We present a 41-year-old woman with Crohn’s disease who developed infliximab-induced systemic lupus erythematosus, manifesting as acute recurrent pericarditis. Initial CMR demonstrated marked pericardial edema and circumferential late gadolinium enhancement (LGE). Infliximab was discontinued and colchicine initiated; recurrence the following year prompted introduction of interleukin-1 blockade (anakinra), resulting in durable remission. Serial annual CMR examinations showed progressive reduction in inflammatory markers: severe LGE and edema (2019), moderate enhancement (2020), mild (2021), trace (2022), and trivial residual enhancement (2024). Echocardiography confirmed preserved biventricular function without constrictive physiology. Following clinical stability, anakinra was safely discontinued with continued remission on colchicine.

This case emphasizes the importance of CMR in tracking pericardial inflammatory activity, informing therapeutic decision-making, and identifying resolution in biologic-induced autoimmune pericardial disease, particularly during de-escalation of immunomodulatory therapy Fig. 1.

**Fig. 1:**
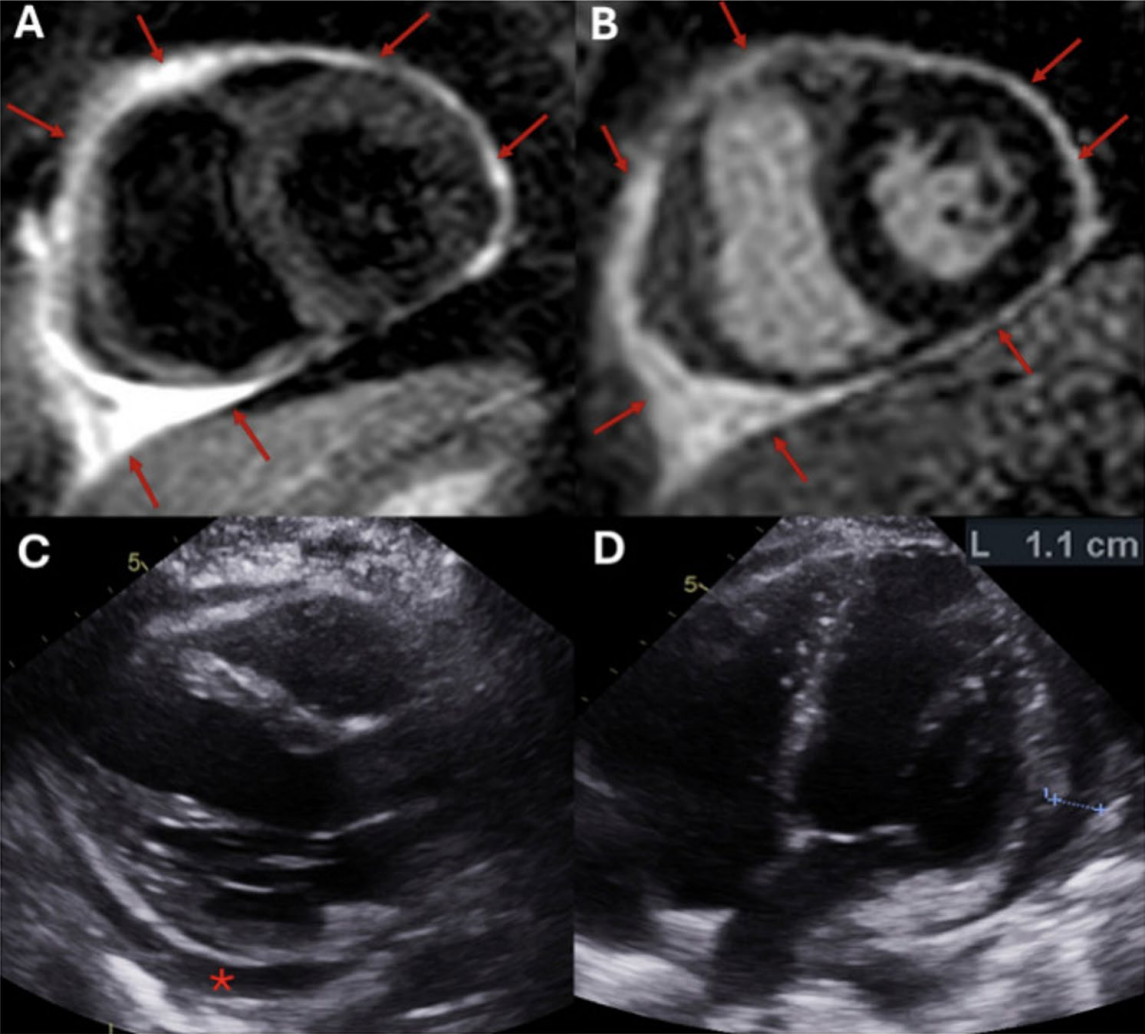
Multimodality imaging findings in active pericarditis with pericardial effusion. Panel **A**: Cardiac MRI T2-STIR sequence showing marked circumferential pericardial hyperintensity consistent with inflammation and edema (red arrows). Panel **B**: Cardiac MRI delayed enhancement sequence demonstration circumferential pericardial late gadolinium enhancement (LGE), indicating active inflammation and fibrosis (red arrows). Panel **C**: Transthoracic echocardiogram (parasternal long-axix view) showing a small circumferential pericardial effusion (asterisk). Panel **D**: Transthoracic echocardiogram (apical four-chamber view) demonstrating pericardial effusion measuring 1.1 cm lateral to the left ventricle (asterisk)

## Data Availability

No datasets were generated or analysed during the current study.

